# Strategies of Asian, Hispanic, and Non-Hispanic White Parents to Influence Young Adolescents’ Intake of Calcium-Rich Foods, 2004 and 2005

**Published:** 2008-09-15

**Authors:** Marla Reicks, Miriam Edlefsen, Dena Goldberg, Garry Auld, Margaret A Bock, Carol J Boushey, Christine Bruhn, Mary Cluskey, Scottie Misner, Beth Olson, Changzheng Wang, Sahar Zaghloul

**Affiliations:** Department of Food Science and Nutrition, University of Minnesota; Food Science and Human Nutrition, Washington State University, Pullman, Washington; Food and Nutrition Services, Carilion Roanoke Community Hospital, Roanoke, Virginia (Institution where work was done: University of Wyoming); Food Science and Human Nutrition, Colorado State University, Fort Collins, Colorado; Family and Consumer Sciences, New Mexico State University, Las Cruces, New Mexico; Foods and Nutrition, Purdue University, West Lafayette, Indiana; Food Science and Technology, University of California, Davis, Davis, California; Nutrition and Food Management, Oregon State University, Corvallis, Oregon; Nutritional Sciences, University of Arizona, Tucson, Arizona; Food Science and Human Nutrition, Michigan State University, East Lansing, Michigan; Kentucky State University, Frankfort, Kentucky; Human Nutrition, Food, and Animal Sciences, University of Hawaii, Honolulu, Hawaii

## Abstract

**Introduction:**

Optimal intake of dietary calcium is critical to prevent osteoporosis later in life, yet most young adolescents do not consume the recommended amount. We describe parental strategies that can influence young adolescents' calcium intake in Asian, Hispanic, and non-Hispanic white households.

**Methods:**

A qualitative research design employed semistructured individual interviews with a convenience sample of mostly female parents self-reported as Asian (n = 48), Hispanic (n = 44), or non-Hispanic white (n = 76) having a child aged 10 to 13 years at home. Interviews were conducted in homes or community centers in 12 states. Interview data were analyzed by using qualitative data analysis software and thematic content analysis procedures.

**Results:**

Parents monitored calcium intake by making calcium-rich foods available, preparing calcium-rich foods, and setting expectations that children would consume calcium-rich foods. As mentors, parents encouraged intake of calcium-rich foods and advised children to moderate or increase intake of specific foods. Although parents perceived modeling of calcium intake as important, some were ambivalent about its effects. We noted minimal differences by racial/ethnic groups and sex of children in reported availability of selected calcium-rich foods at home, parental modeling of intake, and mentoring behaviors.

**Conclusion:**

Our findings suggest that interventions to help parents increase children's intake of calcium should focus on types of foods made available, giving age-appropriate encouragement and advice, and modeling proper intake.

## Introduction

Adequate calcium intake during adolescence is crucial for bone health later in life (1), yet dietary calcium intake of US adolescents is below recommendations and begins to fall during this stage of life. Among children aged 9 to 13 years, calcium intake from food has been estimated to meet only 67% of requirements for girls and 88% for boys (2).

Because calcium intake varies by children's race/ethnicity (3-5), understanding how the family environment influences calcium intake may be particularly important to address the decline in calcium intake as children grow older. Social cognitive theory (SCT) explains human behavior in terms of a triadic, dynamic, and reciprocal interaction between personal factors and influences of behavior and environment (6). Therefore, SCT can serve as a basis to describe the influence of family and household environment on children's eating behaviors.

Parents and caregivers serve as the primary food gatekeepers, controlling the availability of foods in the home (7-9). They determine family meal structure, patterns, and intake. Among children aged 9 to 14 years, increased frequency of family dinners was associated with higher calcium intake (10). Parental expectations for intake of particular foods can also influence nutritional intake (11-13). Parents and caregivers act as mentors and shape children's food-related beliefs, attitudes, knowledge, and preferences through the use of food socialization practices, which include sharing verbal and nonverbal messages about foods and nutrition (14,15).

Parents and caregivers can model intake of healthful foods (11). In one study, mothers who drank more milk also drank fewer soft drinks; their daughters had similar beverage consumption patterns (16). In another study, milk consumption by a significant adult was associated with improved calcium intake for adolescents (17).

Eating behaviors in families are personal and culturally embedded, in particular when feeding children. Cultural influences on parenting in the context of childrearing and risk behaviors have been examined (18-20). For example, Latinos' parental monitoring of smoking behavior was more protective than that of other groups (19). Mexican-American mothers reported more authoritarian parenting than did white mothers (18). However, little is known about the cultural variations in parenting practices involving eating behavior of young adolescents. Cullen et al (21) reported few ethnic differences in food socialization practices, self-efficacy, or parenting style regarding school-aged children's fruit and vegetable consumption. Previous studies of parental influences on children's intake of calcium-rich foods did not include cross-cultural comparisons (16,22), although some researchers have examined factors affecting intake of these foods from the perspective of children from various cultural backgrounds (23,24). Studies that examine parental monitoring and authoritarian parenting (ie, rule enforcement by power assertion) regarding eating behaviors of early adolescents could provide a better understanding of how culture affects intake of calcium-rich foods in families.

We describe strategies of Asian, Hispanic, and non-Hispanic white parents that influence calcium intake by young adolescents (aged 10 to 13 years). Our results have implications for the development of intervention programs tailored to specific needs of parents and their young adolescent children.

## Methods

We employed a qualitative research design using semistructured individual interviews with a convenience sample of 201 parents from 3 racial/ethnic groups: 1) Asian (n = 48), 2) Hispanic (n = 44), and 3) non-Hispanic white (n = 76). Interviews with parents who identified their child as being of mixed race/ethnicity (n = 33) were not included in the data analysis. Researchers from 12 state universities used flyers, word of mouth, personal contact, and e-mail to recruit parents of children participating in church, Scout, 4-H, community athletic, and county nutrition education programs for low-income children. Participants had a child aged 10 to 13 years living in their home and were primarily responsible for buying and preparing food. This study was conducted as part of a US Department of Agriculture multistate research project (W-1003). The university institutional review board for each university researcher approved the study.

Interview questions were developed on the basis of concepts from SCT and from previous studies involving calcium intake among young adolescents (23,24). Questions assessed parental roles regarding children’s consumption of calcium-rich foods and beverages, including monitoring, mentoring, and modeling practices. Questions explored how parents 1) monitored intake by controlling availability, structuring meal patterns, and setting expectations for meals and snacks (for example, “Tell me about your meals together as a family. What do you expect your child to drink with meals and snacks?”); 2) mentored children through teaching, encouragement, and conversations about food and health (for example, “What kind of conversations do you have about food and health with your child? What kinds of things do you say?”); and 3) modeled the desired behavior by consuming calcium-rich foods (for example, “Tell me about a food you think is healthy for your child. How do you get your child to eat it? Do you eat this food? How does this influence your child?”). The initial interview guide was pilot tested with 28 parents by 8 researchers, and results were used to modify questions for continuity and clarity.

Researchers in each state either conducted the interviews or trained others (graduate and undergraduate students and paid program assistants) to serve as interviewers. Training consisted of reading background information (25,26), watching and discussing a videotaped demonstration, and participating in a conference call facilitated by an expert in qualitative methods to review data collection methods. Several researchers also conducted supervised practice interviews with interviewers.

We audiotaped semistructured face-to-face interviews with parents in each state in public settings (eg, restaurants, workplaces, bookstores), community settings (eg, cooperative extension offices, community centers, athletic facilities), or in the homes of parents. Interviews lasted 30 to 90 minutes (average, 60 minutes). Respondents were given $20 to $25 (in cash or gift certificates). Approximately 75% of the interviews were conducted solely in English. The remaining interviews were conducted solely or partly in other languages such as Spanish, Chinese, or Hmong. Demographic information was collected using a short written form before the interview. Interviewers also took notes during the interviews.

Demographic data were analyzed by using SPSS software (SPSS for Windows, release 11.5, SPSS Inc, Chicago, Illinois). Analysis of variance assessed differences in age of children by race/ethnicity and sex, and χ^2^ analysis was used to determine differences in parent/household categorical variables by race/ethnicity.

Interview tapes were transcribed verbatim in each state. Interviews taped in a language other than English were translated by the bilingual interviewer or a translator during the interview or transcription process. Four trained coders used NVivo software (Version 2, QSR International, Doncaster, Victoria, Australia) to code interview transcripts (27). Queries designed to find portions of text that could answer particular research questions were developed and then used to search for and sort the coded transcript segments for further analysis. The segments were sorted by racial/ethnic group and sex of the child. At least 2 researchers independently read the coded and sorted segments from each racial/ethnic group, generated common themes, and identified exceptions by using thematic content analysis procedures (25). The researchers confirmed themes with each other before preparing summaries of results.

## Results

Parents of 168 children in early adolescence were interviewed based on parents' indication of their child's race/ethnicity. Girls made up 51% of the total number of children. The mean (standard deviation) age of all children was 11.6 (1.0) years with no differences by sex or race/ethnicity (data not shown).

Overall, the parents were well-educated. Hispanic parents had lower levels of education and were more likely to have children participating in free or reduced-price school meals than were other groups (Table). Most parents were mothers. Asian parents were more likely than were parents of other racial/ethnic groups to report that English was not the language spoken in the home.

Results are organized by the roles parents described in defining their child's home food environment, with an emphasis on strategies that influence consumption of calcium-rich foods and beverages within each role. Three primary parental roles were described: 1) monitors of intake as a food gatekeeper, controlling food and beverage availability, controlling the structure of meals and snacks, and setting limits and expectations; 2) mentors actively helping children learn about healthful foods and their relationship to health; and 3) models setting an example by modeling personal beliefs and attitudes about the importance of calcium for health.

### Parents as monitors of intake

Parents monitored their children's eating and sought a balance between controlling food choices and permitting independence. The gatekeeper role was seen as a way to encourage healthful eating habits. Parents made many choices about when to intervene in limiting their child's choices.

Parental control emerged in 3 ways. First, parents served as gatekeepers by purchasing foods for home use. Almost all parents said "healthy" foods are available for meals and snacks, although most also allowed less healthful "junk food" items for variety and enjoyment. For example, 1 non-Hispanic white parent said, "I had these bowls filled with apples, oranges, and bananas, and some of my kids will eat things like that when I have them available." Parents primarily decide which foods to purchase, but children's preferences and desires influenced their purchases: "These [dairy] foods are expensive, but my kids like to eat them and I do, too, so we still buy them" (Asian parent).

Second, parents mainly controlled the preparation of meals and snacks, and children were granted varying degrees of responsibility for preparation. Breakfast was characterized by most parents as being "on your own" or "on the run." Several parents supervised their child's preparation and consumption of breakfast, but many families gave children responsibility for choosing, preparing, and eating their own breakfast.

Parents seemed least involved with their child's lunch because most children ate lunch at school, and parents could not oversee this meal. Few indicated they checked with their child about what they had eaten at school. Thus, children exercised more freedom and responsibility in choosing foods for lunch. Some parents gained control over their child's choices by packing a lunch daily. One Hispanic parent said:

I am really concerned because I'm not sure what she is eating there, because they have choices. . . . I'm not sure how much balance or what they're getting is good for them or not. So when they take lunch from the house then it is more controlled.

Parents applied the most control over dinner, mainly because they frequently prepared this meal for their families. All parents said they wanted to eat dinner at home with their families as often as possible: "Our dinners are like our family time so we try to eat together" (non-Hispanic white parent). Another common topic for all parents was foods their child liked and preferred to have served at meals. Parents frequently indicated that their child's preferences influenced what was served for dinner.

Most parents did not actively prepare snacks for their child nor did they closely monitor snacking behavior: "They're getting older now, they kind of help themselves" (Hispanic parent). Snacking patterns were similar among racial/ethnic groups; however, some Hispanic families reported eating dinner shortly after children came home from school.

A third manner of parental monitoring involved expectations that children eat certain foods at meals. Parents tried to balance guiding food choices and allowing independent decisions by their child. A few parents indicated they have no expectations for foods consumed for meals, and children were allowed to eat anything they wanted. Most parents had some expectations for eating particular foods such as meat, vegetables, or rice. Hispanic and non-Hispanic white parents commonly expected their children to at least taste a new or unfamiliar food or to try a little of every food offered at the meal, even if it was only "a bite":

We expect him to at least try it. . . . The other day he thought that Brussels sprouts were nasty and when he tried them, they were good. I thought it was a major milestone. (non-Hispanic white parent)

Several parents emphasized that they do not force their children to eat foods the children dislike. In contrast, a small number of parents spoke of "making" their children eat certain foods they thought were good for them, and many said that children should learn to eat what was prepared for the meal ("This is not a restaurant" and "She eats what we eat"). Several parents expected children to eat everything on their plate, but others allowed children to choose what they wanted to eat but expected them to eat everything they had chosen: "I expect them to eat everything they take, which is what I had to do" (non-Hispanic white parent).

In general, when parents indicated they had no expectations for intake of certain foods at meals, it was because they prepared foods their children would eat or because their child was a "good eater." Vegetables were the food most often mentioned by parents as desirable to finish, followed by meat and fruit. Most parents from all racial/ethnic groups allowed children to choose from several beverages for meals, and many did not have strong expectations for drinking milk with meals: "They can have juice, milk, water, whatever they're thirsty for" (Hispanic parent). "Anyone who wants something gets it themselves. . . Pepsi, juice, water and ice. . . . Milk is in the fridge, and if they want it they can get it" (Asian parent).

A few differences existed between ethnic groups in terms of expectations — only Hispanic parents mentioned that they expected children to eat whatever the parent served them and were more likely to say their child was "a good eater." Only parents of non-Hispanic white girls mentioned that they expected their children to avoid certain foods (eg, carbonated soft drinks, cheese products). Finally, some parents of Asian children mentioned "making," "forcing," and "struggling" to get their children to eat foods they didn't want to eat.

### Parents as mentors

Parents described taking advantage of various everyday situations to talk with their child about different food- and health-related topics. Parents described these as *teachable moments,* which were prompted by activities such as shopping for food, preparing meals, eating and choosing snacks at home, choosing foods when eating out, or refusing to eat specific foods. For example, one parent taught how to make healthy choices while negotiating with her children about choosing a snack:

When they ask for an after-school snack they'll look at the labels and I'll say, you can have a cookie, but let's have something else that's good for you. (non-Hispanic white parent)

Parents talked with girls but not boys about food and health during food preparation, indicating differences in the way parents treat girls. Conversations with children tended to be related to the child's athletic performance or something the child learned about food and health at school.

Some parents felt that they should verbally encourage or remind their child to eat healthfully more often. For other parents, verbal encouragement was less important than their ongoing efforts to establish healthful eating patterns by example:

I never tell her what I expect her to eat. I make available healthy foods, and she makes the right choices more often than not. (non-Hispanic white parent)

In general, topics for discussion focused on the relationship between foods and health. Parents felt it was important to describe reasons for eating specific foods to obtain specific nutrients and health benefits:

Sometimes they prefer to drink something else [besides milk]. And I say, "You need to have strong bones. And you'll grow and you need calcium." (Asian parent)

I tell them when I serve the food, this is for your eyes, this is for your bones and teeth. I try to convince them about the things in the food that are good for them. (Hispanic parent)

Parents were most likely to encourage children to eat more dairy foods and fruits and vegetables.

Parents also encouraged children to use moderation in choosing high-fat, high-calorie foods, described as "junk food," "sweets,"  "fast food,"  and snack foods such as chips and carbonated soft drinks. Some parents focused on improving variety and balance in food choices. "I tell her, OK, you can have that, but you need to have salad first" (non-Hispanic white parent). Parents also discussed the benefits of having a healthy weight, improving sports performance, avoiding illnesses, or developing good habits when young:

I tell them to try and not eat all the fattening stuff. . . . They are going to gain weight, and it's going to be hard to lose. (Hispanic parent)

Parents of Asian children reported a focus on specific foods and benefits such as the need to eat meat for muscles and blood, calcium to grow tall, or fish for the brain. Parents of Hispanic children often mentioned having conversations about food and weight control including healthful snacking. Hispanic parents were also more likely to link the need for healthful eating with a family history of diabetes and heart disease. Although many parents indicated that the purpose of having conversations was to teach or explain concepts, parents of Asian boys used words such as *pressure*, *convince*, *push*, and *lecture*.

### Parents as role models

Most parents said they set an example for their child by eating foods they think are healthful to positively influence their child's eating habits, saying their child "copies"  what they do, or "if they see me try it, they'll try it." For example:

I try to eat [dairy foods] just to show the kids it's good for them because they don't like dairy foods very well. (non-Hispanic white parent)

If I wasn't drinking [milk] then she would feel like she doesn't have to drink it. So since everybody drinks it, she knows that she needs to drink it. (non-Hispanic white parent)

Parents had strong beliefs that calcium is necessary for strong bones and teeth, for protecting from weak bones that result from aging, and for growth. Parents recognized the importance of dairy foods such as milk, cheese, yogurt, and ice cream in meeting calcium requirements, as well as nondairy foods such as soy milk, vegetables (particularly greens and beans), and calcium supplements.

Many parents consumed dairy products daily or tried to include other calcium-rich foods in their daily diet. Most parents said they drink milk and eat cheese; fewer said they eat yogurt. However, many parents who did not consume dairy products set an example for their child by consuming calcium supplements, soy milk, or other calcium-rich foods. Some Hispanic and non-Hispanic white parents spoke of milk as the main beverage with meals or as a traditional beverage. Hispanic parents mentioned using cheese as part of many meals. However, fewer Asian parents consumed dairy products regularly and were, therefore, less likely to model intake of dairy foods. These parents sometimes spoke of other calcium-rich foods they consumed, such as soy milk, greens, or calcium-fortified orange juice.

Several parents also emphasized the importance of exposing children to healthful foods from an early age:

So, when she began to eat the table foods that we eat, I made sure she shared what we had and saw that we enjoyed it, then she would know by example. (Hispanic parent)

A small number of parents felt their example did not influence their child's eating.

He eats what he likes — [sometimes he] just dislikes the food. (Hispanic parent)

Parents of non-Hispanic white and Hispanic boys seemed less certain that their example had an influence on their child's behavior than did parents of non-Hispanic white and Hispanic girls.

## Discussion

We found that the role of parents as monitors of calcium intake involved making foods available by purchasing and preparing them and setting expectations for their consumption. Results from several studies support a relationship between household availability of foods and beverages and intake by adolescents (28,29). Household availability of soft drinks was inversely associated with girls' dairy intake, and serving milk at meals was positively related to boys' dairy intake (30). Calcium intake of adolescents was associated with availability of milk with meals (31). Results from interviews with mothers who had participated in an osteoporosis prevention trial indicated that they used strategies to increase children's calcium intake that also involved making calcium-rich foods accessible to children and considering children's preferences (32).

The parents in our study perceived that they had a strong impact on their children's food intake through meal preparation, especially for dinner, and that children had more independence and control for breakfast (eaten quickly and independently) and lunch (typically consumed at school). Individual interview results with children aged 11 to 18 years (33) confirmed our findings, showing that most adolescents perceived the highest level of control over their food choices for breakfast and lunch and reported interacting with family in various ways to achieve some personal control over the dinner meal.

To a lesser degree, parents in our study monitored their children's calcium intake through setting expectations and rules. Many parents did not have strong rules or expectations about eating particular foods at meals or snacks and often accommodated their children's food preferences.  Most parents allowed their child options, including milk, water, or juice. Few expected that milk would be the beverage of choice with dinner, which was not consistent with results of another study (13), in which 41% of children aged 10 years indicated that their parents always expected them to drink milk.

Parents in our study mentored their children through conversations and encouragement toward healthful food choices, indicating social support and a high level of involvement with their children. Parents often used reasoning to explain the relationship between intake of particular foods and health and relied occasionally on negotiation as a tool to encourage intake of healthful foods. Studies investigating the role of parental encouragement and support on eating behavior of children and adolescents have focused on fruit and vegetable, fat, or calcium intake (8,32,34) and supported an association between this type of parental involvement and desired intake. For example, adolescents reported that encouragement from family was a common and helpful support mechanism for healthful eating (35), and among boys, parental encouragement was positively associated with calcium intake (32).

As children age and become more independent in making food choices, enforcing rules or monitoring the  effects of encouragement becomes more difficult for parents. Several parents in our study indicated that they made greater efforts to encourage healthful food choices when their child was younger but that they had relaxed their vigilance as the child grew older. Intergenerational focus groups indicate that ongoing, harmonious communication between children and parents about healthful eating instilled these values from the time children were young (36). Involvement to promote healthful eating among adolescents is still important and needs to be fostered.

Topics of conversation between children and parents in our study focused on the link between nutrients, food, and health and the need for moderation and balance. Conversations varied greatly among respondents and seemed tailored to particular situations, individual needs, and eating patterns of each child. This finding was encouraging because other researchers have suggested that generic nutrition advice embedded in conversations during family mealtimes may be more easily dismissed as not applicable to individuals (37).

Not all parents in our study consciously modeled consumption of calcium-rich foods, although nearly all parents understood the link between calcium and bone health. Many parents of adolescents do not consume the recommended dairy servings (31), even though parental dairy intake was positively associated with increased dairy consumption among their adolescent children. Several studies have also shown that adolescents' perceptions of parental modeling can influence food intake of adolescents, specifically intake of high-fat snacks and fruits and vegetables (11,30,38). In our study, parents of girls had a stronger perception than did parents of boys of the effects their modeling might have on their children's behavior. This finding suggests a difference in parental strategies according to sex of the child with implications for public health interventions.

A previous study (21) found only minor differences between ethnic groups in social environmental influences on school-aged children's intake of fruit, juice, and vegetables. Child and parent perceptions of parenting style, food socialization practices, food preparation, menu planning, and food purchasing practices were assessed for African American, Hispanic, and non-Hispanic white groups. The findings from our qualitative study also found few major ethnic differences among parental practices and support a commonality across racial/ethnic groups related to parental influences on the home food environment. Knowledge of these similarities among groups is important for practical application in community education programs. This knowledge allows educators to determine the need to tailor educational activities to diverse audiences when developing and delivering education.

We were not able to quantify differences in parental practices or attitudes according to acculturation. Acculturation into US society has been shown to exert negative effects on dietary practices for Hispanics and has been associated with worse health outcomes (39). Negative effects include greater intake of fat and lower intake of fiber, protein, vitamins, and minerals. Acculturation may also be expected to influence attitudes and practices of parents as monitors, mentors, or models regarding adolescents' food intake. For example, in a study with immigrant mothers, attitudes about the importance of family meals varied by ethnic group; Hispanic mothers indicated that family meals were important to maintain health, whereas Asian mothers felt that family meals were important to meet the need to eat familiar foods (40). In another study of parents with young children, less acculturated Hispanic parents were more likely to report using a controlling parenting style regarding physical activity than were more acculturated parents (41).

Our findings have implications for intervention programs based on the parental roles described in our study (Box). To address the parental role of monitor, activities could focus on calcium-rich foods and beverages that could be purchased or prepared for breakfast and snacks according to preferences of children. For the role of mentor, activities could assist parents in generating expectations to share with their children regarding the intake of calcium-rich foods and beverages for meals and snacks based on sugar or fat content. Lastly, in the role of model, activities could focus on behavioral strategies, such as goal-setting and self-regulation activities, to increase consumption of calcium-rich foods and beverages when eating meals and snacks with children.

**Box.** Strategies of Hispanic, non-Hispanic White, and Asian Parents for Influencing Adolescents' Calcium Intake

**Role**	**Parental Activity**
Monitor	1. Purchase and provide calcium-rich foods and beverages according to preferences of children. 2. Make available breakfast and snack foods that are good sources of calcium. 3. Set expectations for intake of calcium-rich foods and beverages from meals and snacks, using appropriate parental control.
Mentor	4. Take advantage of teachable moments to emphasize health benefits from calcium-rich foods and beverages. 5. Encourage moderation in the intake of high-fat, high-sugar foods as sources of calcium. 6. Provide nutrition advice from an individual perspective instead of providing generic advice. 7. Teach food preparation skills specifically for breakfast, lunch, and snacks.
Model	8. Consume calcium-rich foods and beverages (dairy and nondairy) at meals and snacks with children. 9. Continue to model intake of calcium-rich foods and beverages throughout the life stages of children.

The use of a convenience sample is a limitation of our study. Therefore, our findings may not represent the full range of attitudes and practices found among the ethnic groups studied. However, data were collected across 12 states, bringing more diverse perspectives than might be found in a single geographic area. The qualitative nature of this study does not allow for determination of a direct causative relationship between specific parenting practices and young adolescents' intakes of calcium-rich foods and beverages, but our results suggest patterns within households that can be further explored and quantified by future research.

## Figures and Tables

**Table.** Parent/Household Characteristics by Child's Race/Ethnicity, in Sample of Parents (N = 168) Interviewed About Their Influence on Child's Intake of Calcium, 2004 and 2005

**Parent/Household Characteristics**	Parents of Hispanic Children(n = 44) n (%)	Parents of non-Hispanic White Children (n = 76) n (%)	Parents of Asian Children (n = 48) n (%)
**Sex of interviewed parent**			
Female	41 (93)	72 (95)	45 (94)
Male	3 (7)	4 (5)	3 (6)
**Parent's race/ethnicity**			
Hispanic	42 (95)	0	0
Non-Hispanic white	2 (5)	76 (100)	0
Asian	0	0	48 (100)
**Parent's education^a^**			
High school or less	11 (25)	6 (8)	13 (27)
Some college	22 (50)	13 (17)	5 (10)
College graduate	10 (23)	57 (75)	29 (60)
**Child receives free or reduced-price school lunch **			
Yes	29 (66)	9 (12)	19 (40)
No	15 (34)	67 (88)	29 (60)
**Language usually spoken in the home**			
English	22 (50)	76 (100)	16 (33)
Spanish	22 (50)	0	1 (2)
Asian language^b^	0	0	31 (65)
**Days/week child cares for self after school**			
Never	33 (75)	54 (71)	33 (69)
1	11 (25)	22 (29)	15 (31)

a Education data are missing for 1 parent from each subgroup.

b Chinese, Korean, Hmong, Vietnamese, or Thai.
